# Size-Exclusion Particle Separation Driven by Micro-Flows in a Quasi-Spherical Droplet: Modelling and Experimental Results

**DOI:** 10.3390/mi12020185

**Published:** 2021-02-12

**Authors:** Giovanni Marinaro, Christian Riekel, Francesco Gentile

**Affiliations:** 1 Faculty of Mechanical Science and Engineering, Institute of Process Engineering, Technische Universität Dresden, 01062 Dresden, Germany; 2 Institute of Fluid Dynamics, Helmholtz-Zentrum Dresden-Rossendorf (HZDR), 01328 Dresden, Germany; 3 The European Synchrotron, ESRF, CS40220, CEDEX 9, F-38043 Grenoble, France; riekel@esrf.fr; 4 Department of Experimental and Clinical Medicine, University of “Magna Graecia”, 88100 Catanzaro, Italy; francesco.gentile@unicz.it

**Keywords:** droplet-based microfluidics, particle separation, superhydrophobic surfaces, particle image velocimetry, modelling of drying droplets

## Abstract

Aqueous solution droplets are supported quasi contact-free by superhydrophobic surfaces. The convective flow in evaporating droplets allows the manipulation and control of biological molecules in solution. In previous works, super-hydrophobic drops on nano-patterned substrates have been used to analyze otherwise undetectable species in extremely low concentration ranges. Here, we used particle image velocimetry (PIV) for studying the flow field in water droplets containing polystyrene particles on a pillared silicon super-hydrophobic chip. The particles describe vortex-like motions around the droplet center as long as the evaporating droplet maintains a spherical shape. Simulations by a Finite Element Method (FEM) suggest that the recirculating flow is due to the temperature gradient along the droplet rim, generating a shear stress. Notably, the characteristics of the internal flow can be modulated by varying the intensity of the temperature gradient along the drop. We then used the flow-field determined by experiments and an approximate form of the Langevin equation to examine how particles are transported in the drop as a function of particle size. We found that larger particles with an average size of 36 μm are preferentially transported toward the center of the substrate, differently from smaller particles with a 10-fold lower size that are distributed more uniformly in the drop. Results suggest that solutions of spherical particles on a super-hydrophobic chip can be used to separate soft matter and biological molecules based on their size, similarly to the working principle of a time-of-flight (ToF) mass analyzer, except that the separation takes place in a micro-sphere, with less space, less time, and less solution required for the separation compared to conventional ToF systems.

## 1. Introduction

Aqueous solution droplets supported by pillared superhydrophobic surfaces (SHSs) provide quasi wall-free microfluidic environments for soft and biological matter solutes [1,2]. The initial contact angle of evaporating droplets remains constant until the wetting transition, resulting in pinning and the formation of coffee-ring type residues [2,3,4]. This is of great interest for the analysis of soft and biological matter solutions, molecular assembly [2,3,4,5], sensing [6,7], and controlled deposition [8]. Micro- and Nanopattered surfaces are also of great interest in bioengineering for the mechanical interactions and adhesion of cells [3]. The convective flow in evaporating droplets allows maintaining a homogeneous distribution of solute and concentrating even ultra-dilute solutions [4].

Notably, the convective flows that are reported to develop in a slowly evaporating droplet can be possibly used as a mechanism to separate species with a different size, shape, or charge, contained therein. Resolving complex mixtures in solution is crucial in a variety of different fields, such as agri-food, environmental pollution, biology, biomedical engineering, the study of bio-molecular interaction, and association dissociation processes at the single molecular level [9]. Among the several different techniques that exist for separating particles in solution, size-exclusion techniques and hydrodynamic chromatography are the most promising [10]. Size exclusion techniques imply injection of the sample mixture through porous media, where the pore size can be finely tuned. Then, particles larger than the average pore size are retained or delayed with respect to smaller particles that can take shorter paths toward the collecting duct of the system. Nevertheless, since pores in a porous medium are often randomly distributed, with multiple paths connecting the inlet to the outlet of the system, size-exclusion particle separation carried out though this method cannot achieve high resolution. To improve the resolution of particle-separation techniques, externally generated flow fields are combined with percolating media. In the method, called hydrodynamic chromatography, separation is accomplished by both hydrodynamic forces and porous sieves. The combination of the two result in enhanced separation efficiency. Remarkably, hydrodynamic chromatography is susceptible of miniaturization. The flow fields necessary to particle transport can be generated in microfluidic channels or chambers, while porous media can be micro-fabricated or nano-fabricated as in Reference [11]. The flow streams arising in a millimetric drop can represent an alternative to conventional microfluidic driven fluid flows. Because of the curvature at the liquid-air interface and of the temperature gradients across that interface, the fluid elements in a drop move and a deformation through the complex, but still predictable patterns. These convective flows, coupled to diffusion [12,13], are those components that may replace material filters of conventional size-exclusion or chromatography techniques enabling particle separation.

The flow field in evaporating droplets on SHSs is, however, not well understood as simulations based on equations of fluid mechanics do not agree on a coherent model. According to one simulation, an evaporation-induced concentration gradient at the interface results in the Rayleigh convection [14,15]. Other work assumes that evaporative interface cooling establishes a non-uniform temperature gradient along the droplet interface resulting in a convective recirculating flow [16]. There are, however, currently limited experimental data on evaporating droplets on SHSs supporting specific models. Cellular flow patterns observed by particle image velocimetry (PIV) [17] for salt solution droplets support Rayleigh convection but were obtained for wetting and hydrophobic surfaces [14,15]. Here, we report on an experimental study of convective flow in evaporating water droplets on SHSs by PIV combined with simulations based on the finite element method (FEM). Results of the analysis and the fluid flow field determined experimentally were then used to estimate the transport of micro-particles in the drop. We observed that particles accumulate in different regions of the drop and the substrate depending on particle size. A size-dependent trajectory in a curved domain as a drop can be used to develop a strategy for particle separation, identification, and detection, as explained in the rest of the paper.

## 2. Experimental Design and Model

### 2.1. Fabrication of Nano-Patterned Super-Hydrophobic Surfaces

**Figure 1:**
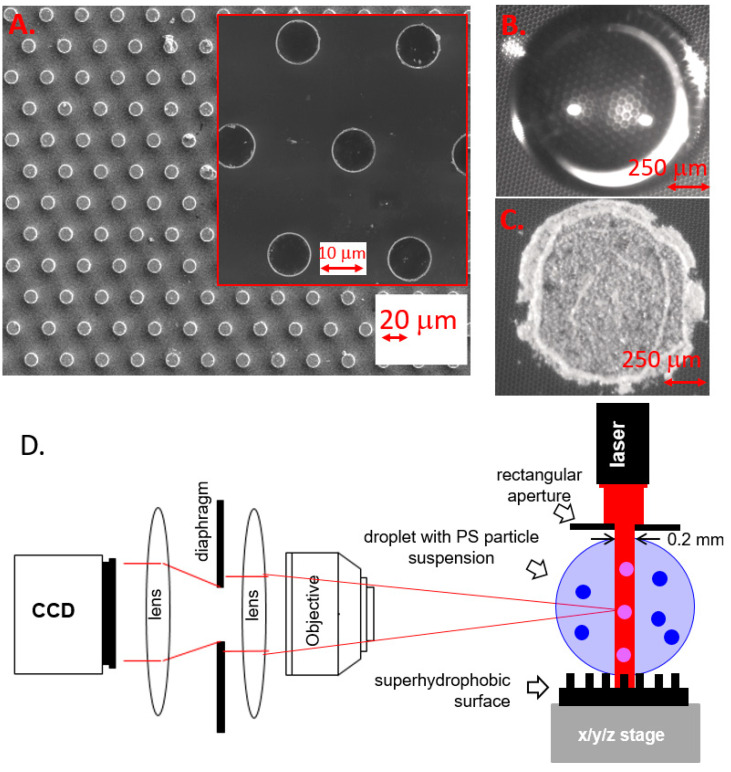
Scanning Eelectron Micrograph image of the pillared surface and zoom in an onset (**A**). Top view optical image of the drying droplet on the Superhydrophobic SurfaceS (**B**). Residue of polystyrene particles after evaporation (**C**). Particle image velocimetry (PIV) setup and speckle imaging. The x/y/z stage allows positioning the droplet relative to the sheet-like $0.2\times 5.6{\;\mathrm{mm}}^{2}$ laser beam (**D**).

We used commercial 2 inches <100> silicon wafers. Pillar patterns were generated by photolithography using a photomask consisting of a periodic, hexagonal pattern of pillars of 10 μm in diameter, and 30 μm in pitch (Figure 1A), according to a method described in Reference [18]. The photomask consisted of a chrome-coated quartz plate designed to optically transfer the pattern to the wafer. The drawing was transferred to a laser mask writer and then printed in a layer of photoresistance coated onto the photomask plate. The patterns were developed over the opaque chrome and the chrome was etched away where the resistance was clear. After the completion of the etch process, the remaining photo-resistance was removed and the plate was cleaned. A positive photo-resistance (S1813) was spin-coated at 4000 rpm and then baked at 90 °C for 180 s over a hot plate. A UV lamp exposure of 15 s at 260 W was applied in a soft contact mode using a mask aligner and the resistance was developed using MF-319 developer for 1 min. We deposited about 5 μL (about 2 mm in diameter) droplets of an aqueous solution containing 5 wt % monodisperse (CV: 1.0%) polystyrene (PS) particles of 9.98±0.32 μm diameter (σ=0.10) with $\rho =1.03\;g/{\mathrm{cm}}^{3}$ (microParticles GmbH: PS-R-10.0) by a manual syringe on the Superhydrophobic Surface (SHS) at room temperature (r.t.) (Figure 1B). For a particle tracking of a larger particle, we deposited 5−μL (about 2 mm in diameter) droplets of an aqueous solution containing 5 wt% monodisperse (CV: 0.8%) polystyrene (PS) particles of 36.03±0.29 μm diameter (σ=0.10) with $\rho =1.03\;g/{\mathrm{cm}}^{3}$ (microParticles GmbH: PS-R-36.0) by a manual syringe on the SHS at room temperature (r.t.). The contact angle was determined after droplet deposition as ϑ~150° by a CAM 101 contact angle measuring system (KSV Instruments Ltd, Helsinki, FI, USA). The total droplet evaporation time resulting in a residue (Figure 1C) was ~60 min. Instead of hollow spherical residues observed for solute molecules with sufficient cohesion in the solid state, we observe a coffee-ring type residue observed for low concentration solutions [19]. This morphology can be attributed to the collapse of an intermediate spherical residue [1].

### 2.2. Particle Image Velocimetry (PIV) Analysis of Fluid Flows in an Evaporating Microliter Drop

**Figure 2:**
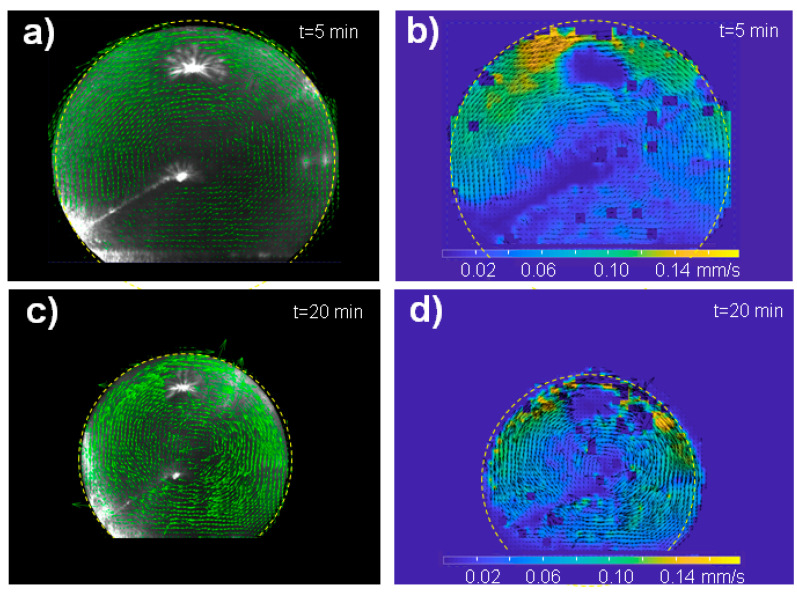
Particle image velocimetry (PIV) analysis of the water droplet loaded with polystyrene (PS) particles evaporating on an superhydrophobic surface (SHS). (**a**,**c**) Vector flow field of droplet at t=5 and 20 min after the start of evaporation. The limit of the droplet is schematically indicated by a dashed yellow circle. The local magnitude of the vortex-like flow is proportional to the length of the vectors. (**b**,**d**) Heat maps of velocity magnitude distribution (in mm/s) overlaying the vector distribution at t=5 and 20 min. Note the change in vector orientation for the two evaporation times.

The PIV setup is shown schematically in Figure 1D. We used a Mitsubishi laser diode of λ=660 nm with 120 mW power and a pinhole size of 5.6 mm. Other parameters can be found in (www.thorlabs.us/thorproduct.cfm?partnumber=ML101J27 (accessed on 23 May 2015)). The laser beam was defined by a $5.6\times 0.2{\;\mathrm{mm}}^{2}$ aperture reducing the power to <2.7 mW (~2%). A significant contribution of the red laser beam to the convective flow is excluded in view of its wavelength in the water window and its low power. Complex convective flow patterns were, however, observed for a higher power laser (0.1–1 W) of λ=532 nm focused to a 0.6−mm spot and an increased absorption by using an oil droplet [12]. Image frames were recorded during evaporation by a DALSA DS-21-02M30 Charged-Coupled Device (CCD) camera with 800×600 pixel resolution for 2×2 binning, set at 10 image frames/s. A relay lens system with a focusing objective provided a full view of the CCD camera on the droplet. The optics was set to obtain a resolution of 200 pixels/mm. A dedicated MATLAB script was written (G.M.) to communicate with the camera. The calibration was performed by recording a transparent ruler at the focal plane. A sequence of image frames was recorded up to an evaporation time of t=20 min. MATLAB (R2017b, MathWorks) PIVlab, which is a particle image velocimetry (PIV) tool [20], was used to analyse the frames. The vector flow field obtained after PIV analysis based on a region of interest (ROI) of the image is shown in Figure 2a–d.

### 2.3. Finite Elements Method (FEM) Simulations of Recirculating Marangoni Flows in a drying droplet upon a Superhydrophobic Surface

**Figure 3:**
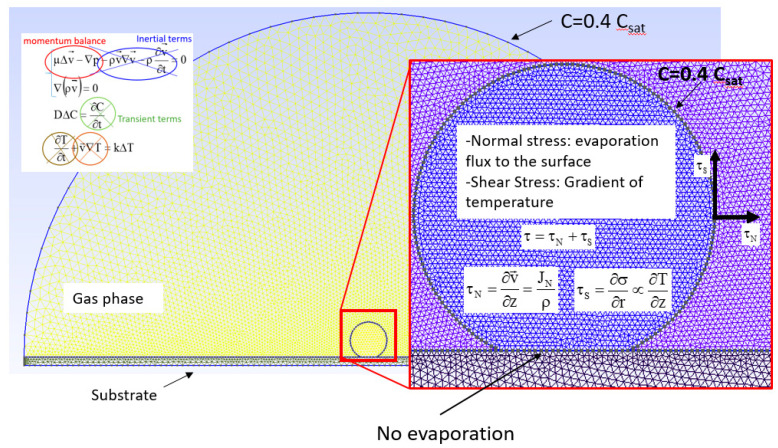
Illustration of the model and boundary conditions.

The partial differential equations, which are solved in the model, are diffusion, Navier-Stokes, and heat transfer. Diffusion describes the vapor concentration of the drying droplet. Navier-Stokes is solved to derive the velocity field inside the droplet and heat transfer resolution is implemented to derive the temperature around the droplet. Consequently, the gradient of temperature at the liquid-gas interface is between the droplet domain and the external environment. Some approximations are then taken into account (for more details, we refer to Table 1 and Figure 3).

Boundary conditions for vapor diffusion are set as follows: no evaporation flux (Neumann Condition) through the support underneath, saturation vapor concentration was set at the interface between the droplet and the air, and the vapor concentration at room temperature (humidity = 40%) was set at the boundary of an external environment at a distance of about 40 mm (Dirichelet boundary conditions). For Navier-Stokes equation boundary conditions, two contributions are considered: evaporation flux to the surface giving a stress condition normal to the droplet interface and gradient of temperature giving a stress condition tangential to the droplet interface (Figure 3). Further details are provided in Section 2.3.2.

#### 2.3.1. Mathematical Background

The 2D diffusion equation is expressed as:

(1) $$D\Delta C=\frac{\mathrm{dC}}{\mathrm{dt}}$$

where D is the diffusion constant of vapor and C is the vapor concentration. Variables, units, and values used for FEM simulation are reported in Table 2. Values for thermal conductivity of droplet, substrate, and air were taken from Reference [16]. For a root squared displacement of 1 mm of water molecules in the vapor phase, diffusion takes place in 0.02 s. The evaporation time of a 1−μL aqueous droplet is ~1000 s as $\rho^{\mathrm{liquid}}>\rho^{\mathrm{vapour}}$. The evaporation is, therefore, much slower than diffusion. The transient terms of the diffusion equation can be neglected by assuming that a concentration gradient is established instantaneously at the interface. The diffusion equation can be, therefore, approximated as:

(2) DΔC=0

where D is the diffusion coefficient in air and C is the vapor molar concentration.

The Navier-Stokes equation is expressed as:

(3) $$\mu \Delta \vec{v}-\nabla p-\rho \;\vec{v}\nabla \vec{v}-\rho \frac{\partial \vec{v}}{\partial t}=0.$$

where the constants ρ and μ are, respectively, the density of the liquid phase and the viscosity, while the variables v, p, and t indicate, respectively, the velocity vector, the pressure, and the time.

For the aqueous suspension, we assume an incompressible Newtonian fluid and the continuity equation is approximated as:

(4) $$\rho \nabla \left(\vec{v}\right)=0.$$

The Reynolds number is defined as:

(5) Re=ρv¯rR/μ,

where μ is the viscosity of the liquid environment, R indicates the radius of the contact area of the droplet with the substrate, and v_r_ is the mean velocity of the flux along the direction parallel to the substrate. Based on the values in Table 2, we estimate a low value of Re~0.003, implying that the contribution of the inertial forces can be neglected and indicating the presence of lamellar flow. The heat transfer is expressed as:

(6) $$\frac{\partial T}{\partial t}+\vec{v}\nabla T=k\Delta T,$$

where T is the temperature and k is the thermal conductivity.

#### 2.3.2. Boundary Conditions

Dirichlet and Neumann boundary conditions [21] were applied to the diffusion equation and Navier-Stokes equations assuming saturation vapor concentration ($C_{\mathrm{sat}}$) at the boundary layer and a vapor concentration of $C_{v}=0.4\;C_{\mathrm{sat}}$ outside the boundary layer (Supplementary Material, Figures S1 and S2). Flux velocity in the droplet bulk ($v_{\mathrm{int}}$) is assumed to be due to normal and shear stresses. We also assume for the flux velocity along the substrate $\left(v_{\mathrm{sub}}\right)$ the absence of fluid slip.

#### 2.3.3. Numerical Model Implementation

The numerical model is implemented in a program written in a MATLAB code based on the differential equations for 2D diffusion, Navier-Stokes flow, and heat transfer (author G.M.) [1]. A transfer of the program to the open source software GNU Octave is currently ongoing (G.M.). FEM analysis was performed for a mesh of triangular elements distributed over a 5−μL droplet with ϑ=155° and its surrounding vapor phase. The mesh of 6152 elements across the droplet and 43,854 elements for the vapor domain (Supplementary Material, Figure S1b,c) was created using the open source Gmsh software [22]. A MATLAB (R2017b, MathWorks) m-file containing the ASCII code was generated to parse the mesh file generated by Gmsh and import nodes, elements, physical domains of droplet, a silicon chip (500−μm thick), and surrounding environment in MATLAB (R2017b, Mathworks). The droplet is considered to be in an open room. However, the limit of the air domain is a spherical cap with a radius 20 times the interface radius (Ri) of the droplet. We suppose that, up to a distance of 20·Ri, the variation of the humidity concentration as well as the temperature variation are negligible, so the humidity is kept at a value of 40% as set at the boundary of the spherical cap. The heat equation is solved by setting the thermal conductivity in the physical domains: water, substrate, and air phase. This applies in the assembly matrix, which is derived by the variational method of Galerkin [23]. A temperature of 21 °C was set at the substrate and the environment boundary. The latent heat of water evaporation (Q) was included as an energy source at the droplet-air interface (Supplementary Material, Figure S1c). The humidity level was set at 40%. The mesh-layer is defined as shown in Supplementary Material (Figure S1c), implying that a layer adjacent to the interface is a few μm distant from the water-air interface. The temperature variation along the droplet air-interface was then computed and introduced as a boundary condition to solve the Navier-Stokes equation and the convective flow inside the droplet. Prior to this, the diffusion equation was solved to determine the normal stress boundary condition for the Navier-Stokes numerical resolution, as described before. For the diffusion equation, the air phase was the sole physical domain considered for the resolution as well as the liquid phase (droplet) for the Navier-Stokes equation. The combination of the gradient of temperature and the normal stress given by the evaporation flux was applied as a boundary condition at the droplet-air interface. With regard to the interface of solid-liquid, no flux was set.

### 2.4. Numerical Solution of the Langevin Equation and Solute Distribution in a Drop

We used the Langevin equation [24,25,26] to determine the distribution of a trace in a slowly evaporating droplet.

(7) $$m\frac{\partial u}{\partial t}=6\pi \mu a\left(K_{p}u-K_{f}v\right)+F_{E}+F_{B},$$

where v is the unperturbed fluid velocity determined by experiments, a is the particle radius, and m is the particle mass. In Equation (7), u is the unknown velocity vector of the particle. The first term on the right-hand side of Equation (7) represents the hydrodynamic drag on the particle. In Equation (7), $F_{E}=0$ is the electrostatic force, while $\left|F_{B}\right|=\varsigma \sqrt{12{\pi a\mu K}_{b}T/\Delta t}$ is the Brownian force. Moreover, ς is a Gaussian number with zero mean and unit variance, $\mu =10^{-3}\;\mathrm{Pa}\;s$ is the viscosity of water, T=298 K is the temperature of the system, and Δt is the discrete time step of the simulation specified elsewhere. $K_{p}$ and $K_{f}$ are diagonal matrices describing the additional hydrodynamic hindrance associated with interactions between the particle and the system boundaries set to zero. For the unperturbed fluid velocity, v, we used the Marangoni flow velocity determined through the particle image velocimetry (PIV) techniques described in a separate experimental section. The fluid flow in a small droplet of water sitting on a super-hydrophobic surface is reported in Figure 2 and replaces the term v in Equation (7) and everywhere in the work. Equation (7) was solved using a numerical scheme [13]. The simulations are forward Euler integrations of the finite-difference equations resulting from discretization of the diffusion and convective operators as in References [27,28]. The initial mesh consists of N=400 grid points. The time step is $\Delta t=10^{-3}\;s$. Initially, the entire system was placed in the initial condition, where 500 identical particles are distributed uniformly within the domain. The system was then integrated over 10,000 time steps and images were saved at specific time points. In all cases, the initial disturbance propagated outward from the initial position to the border of the drop. We found that the radial distribution of solute inside the drop depends on the size and charge of the dislodged particles and this is described in the following in the paper. For solving the equations, we used the Dirichlet condition at the borders, whereby the concentration of the particles is identically zero. This implies that the analysis is valid for the time that the initial perturbation takes to spread over the entire grid. The propagation of the solute was found to depend on the convective flow within the droplet, with the leading edge of the perturbation moving unsteadily with time. The propagation velocity of the initial perturbation and, thus, the dynamic response of the system depends on the parameter values.

## 3. Results

### 3.1. Experimental Analysis of Fluid Flow Fields in the Drop

We deposited about 5−μL volume water droplets containing a suspension of 10−μm diameter polystyrene (PS) particles on a pillared silicon superhydrophobic chip (Figure 1a–c) [18]. In view of the similar density of particles $(\rho =1.03\;g/{\mathrm{cm}}^{2}$) and surrounding liquid, we assume an unperturbed aqueous system. A central sheet of the droplet was illuminated by a red laser beam allowing observing speckles from the polystyrene (PS) particles by a Charged-Coupled Device (CCD) camera. An optical relay system coupled to the CCD camera provided a full view of the droplet (Figure 1d). The dynamics of particle movement for a single droplet in the superhydrophobic state was probed by image sequences with 10 frames/s for 20 min. This compares to a total droplet evaporation time up to residue formation (Figure 1c) of ~167 min at a humidity level of ~40%. The particle dynamics was determined using MATLAB (R2017b, MathWorks) PIVlab tool [20]. The particles perform vortex-like motions around the center of the droplet with the magnitude of flow vectors increasing from the center to the rim (Figure 2a–d). Internal motions of up to about 0.15 mm/s that last as long as the droplet shows a superhydrophobic contact angle ϑ≥150°. The heat maps reveal an increased magnitude of flow at the upper rim of the droplet (Figure 2b,d). A central recirculating flow is observed throughout evaporation (Supplementary Material: Video S1), which does not correspond to the plume-like convective flow, assumed to be generated by a concentration gradient due to evaporation from the interface.

Pradhan et al. studied evaporating droplets of about 1 mm in diameter for aqueous NaCl solutions of different concentrations and they exclude that convection is due to thermal effects and Marangoni flow [14]. According to Pradhan et al., the principal contribution to internal flow is due to buoyancy. They claim that impurities on the surface change the convection flow. The main difference in our PIV measurements is that we observed a central recirculating flow, which does not match Pradhans/Kangs results. The recirculary flow is based, however, on the temperature gradient induced by evaporation and cooling of the droplet. This convective flow resembles a Marangoni flow and serves for transporting material to the interface. The maximum velocity observed during this recirculating flow is 0.16 mm/s.

**Figure 4:**
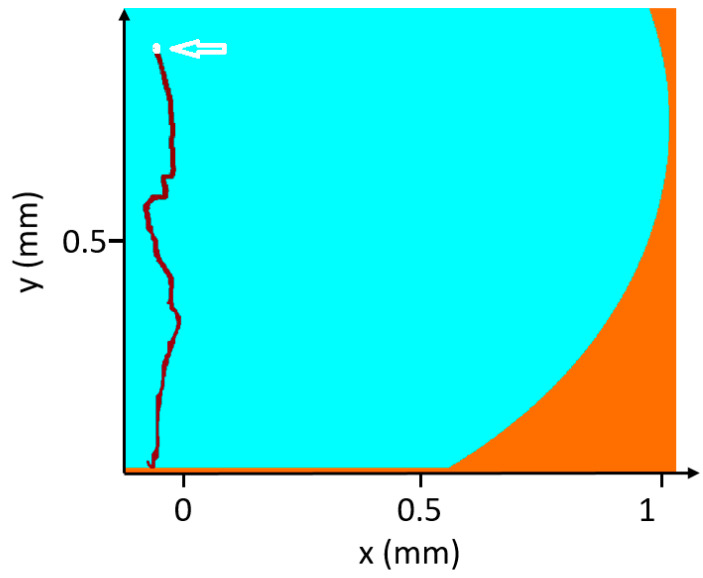
Trajectory of a large polystyrene particle in the convective flow field in the drop.

PIV measurements were conducted to track the path of polysterene particles (size of 36 μm) added to water solution during evaporation and to derive information on the trajectory. Figure 4 shows the trajectory of a single 36-μm particle superimposed in a white color. The starting position of the particle taken after 2 min. Therefore, the initial position is considered at about x_0_ = 0.05 mm, y_0_ = 0.89 mm, marked with a white arrow, and the final position at the bottom is reached after about 20 s. The trajectory of the particle was calculated from subsequent PIV images during the evaporation of the droplet using a method written in MATLAB (R2017b, MathWorks) by G.M., which includes the applications of image processing algorithms and a particle tracking method (for more details, contact G.M.). Particle tracking was limited to larger particles. However, as shown in the video of Supplementary Material, smaller particles continue circulating after 2 min. The recirculation goes on for a longer time until the volume of the drying droplet is further shrunk.

### 3.2. FEM Simulations of Fluid Flow Fields in the Drop

The convective flow was simulated by a FEM approach based on differential equations for 2D diffusion, Navier-Stokes flow, and heat transfer. Physical domain anddiscretization of the domain are discussed and shown in Section 2, Figure 3, and Supplementary Material (Figure S1a and References [1,29,30,31]). Variables, units, and values used for the simulations are reported in Table 2. We approximated the diffusion equation by DΔC=0 where D is the diffusion coefficient and C is the vapor concentration. The transient terms were neglected assuming that a concentration gradient at the interface is established instantaneously. The flow field in the droplet is described by Navier-Stokes differential equations considering a mass and momentum balance $\mu \Delta \vec{v}-\nabla p-\rho \vec{v}\nabla \vec{v}-\rho \left(\partial \vec{v}/\partial t\right)=0$ where μ is the viscosity, v is the velocity, and p is the pressure (Table 2). For diluted aqueous solutions, an incompressible Newtonian fluid and lamellar flow are assumed to result in the approximation of $\rho \nabla \left(\vec{v}\right)=0.$ The contribution of inertial forces can be neglected as the Reynolds number Re=(ρv¯rR)/μ is low ($\sim 10^{-3}$). The heat transfer $\partial T/\partial t+\vec{v}\nabla T=k\Delta T$ is approximated for ΔT=0. Boundary conditions applied to diffusion and Navier-Stokes equations are discussed in Section 2. The differential equations used and their approximations are also discussed in more detail in Reference [1].

**Figure 5:**
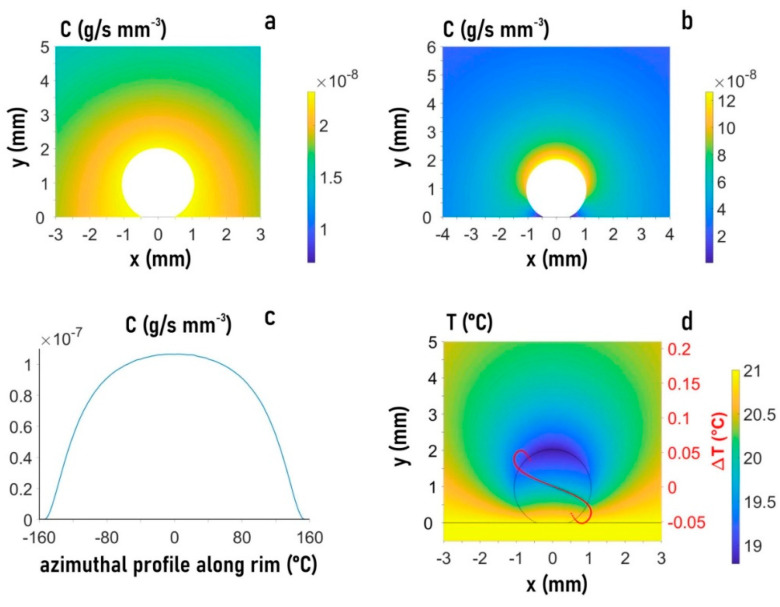
Finite element method (FEM) analysis of the evaporating droplet and its environment for 21 °C and 40% humidity. (**a**) Heat map of vapor concentration based on parula color map array (also **b**,**d**. (**b**) Heat map of the evaporation flux. (**c**) Evaporation flux along the rim of the droplet. (**d**) Heat map of temperature distribution. Overlay of temperature gradient curves at the rim (ΔT) for central recirculating flow (solid red curve) and recirculating flows in two hemispheres (dotted yellow curve). Vertical axis: ΔT (°C), horizontal axis: (mm). The same as for the droplet.

We performed a FEM simulation for a mesh of triangular elements distributed over the droplet and surrounding vapor phase (Supplementary Material, Figure S1a–c). The vapor concentration ($C_{v}$) around the evaporating droplet reveals a homogeneous saturation layer ($C_{\mathrm{sat}}$) decreasing at an external boundary to $C_{v}=0.4{\;C}_{\mathrm{sat}}$ (Figure 5a). The evaporation flux from the droplet shows anisotropy, disappearing at the SHS surface (Figure 5b,c). Cooling induced by evaporation results in a temperature gradient along the rim (Figure 5d). The boundary flow in the two hemispheres of the droplet (τ) is determined by two contributions: the evaporation flux normal to the droplet surface generating a stress normal to the droplet ($\tau_{n}$) and a temperature gradient ($\tau_{s}$) via $\tau =\tau_{n}+\tau_{s}$. The shear stress developing at the interface is assumed to be proportional to the temperature variation. Details on the implementation of the boundary conditions are provided in Reference [1].

**Figure 6:**
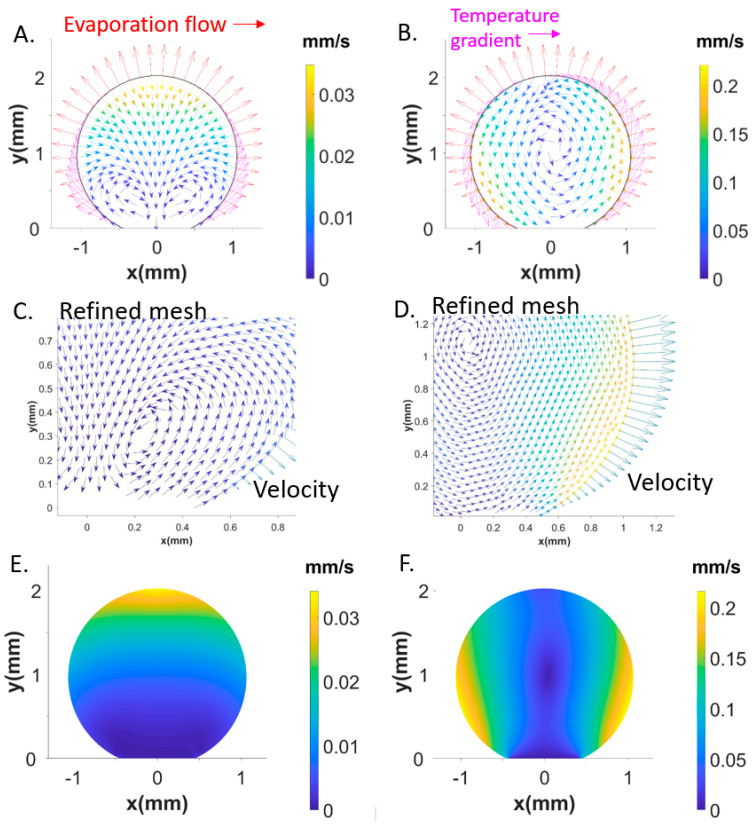
Recirculating flows in the two hemispheres of the droplet revealed by flow vectors. Evaporation flux vectors normal to rim. Tangential stress vectors in pink (**A**). Central recirculating flow (**B**). Recirculating flows in one hemisphere and central recirculating flow shown with a refined mesh (**C**,**D**). Heat map of magnitude of flow vectors for recirculating flow in two hemispheres (**E**). Heat map of magnitude of flow vectors central recirculating flows (**F**).

We explored the boundary conditions for the experimentally observed central recirculating convective flow (Figure 2a–d). FEM simulations reveal a dominating influence of the temperature gradient $\tau_{s}$. Negative $\tau_{s}$ values along the rim shown by the red curve in Figure 5d result in a flow field and magnitude in flow velocity vectors (Figure 6A–F) agreeing rather well with Particle Image Velocimetry (PIV) results (Figure 2b,d). The shear stress generated by $\tau_{s}$ is about 100 times higher than the evaporation-induced stress $\tau_{n}$. The simulations reveal that smaller $\tau_{s}$ values modify the central recirculating flow field into recirculating flows in the two hemispheres of the droplet and reduce the magnitude of flow vectors (Figure 6A,C,E). The red temperature gradient in Figure 5d results about factor 10 smaller flow vectors as compared to the central recirculating flow (Figure 6B,D,F) at the scale of previous simulations [16]. Thus, PIV reveals a central recirculating flow for evaporating droplets on a super-hydrophobic chip, differing from a pure Rayleigh convection observed for droplets on hydrophobic and wetting surfaces [14,15]. FEM simulations do not support a significant concentration gradient at the rim [14] as origin of the central recirculating flow but rather a thermal gradient $\tau_{s}$ requiring a higher shear stress than previously assumed [16].

### 3.3. Solute Transport in the Droplet and Size-Dependent Particle Separation

**Figure 7:**
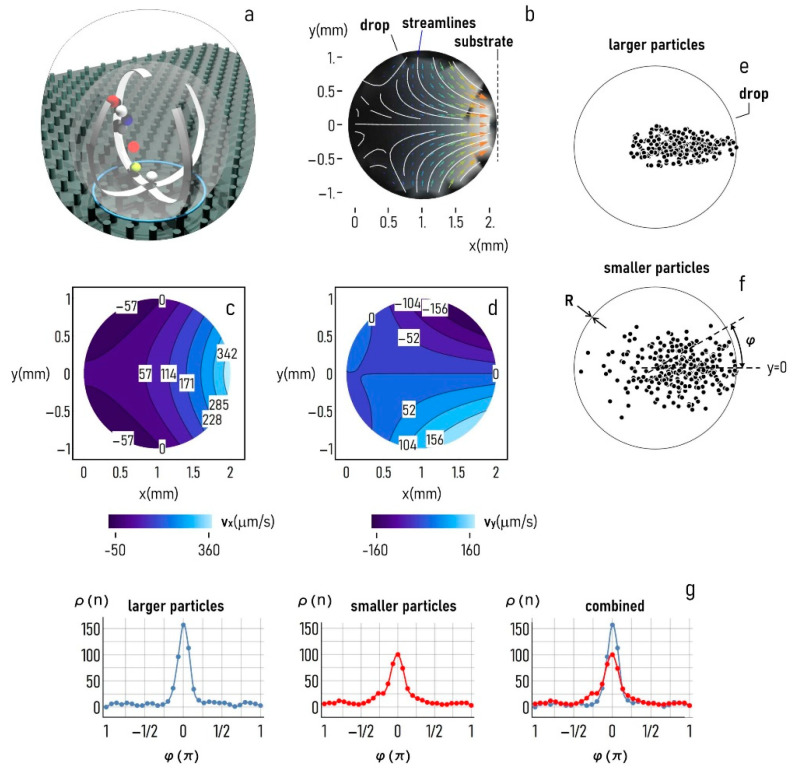
The substrate used in the study uses super-hydrophobic surfaces for maintaining solutions in a quasi-spherical shape (**a**). Because of its curvature and a temperature gradient, in the drop, develop convective flows with characteristic streamlines reported in (**b**) and values of velocity along the horizontal (x) (**c**) and vertical (y) (**d**) direction that vary between 0 and a maximum of ~350 μm/s for the x coordinate and of ~150 μm/s for the y coordinate. The flow fields determined experimentally were used to estimate the transport of large (36 μm–(**e**)) and small (3 μm–(**f**)) particulates within the droplet. The particle density plot against the position in the drop reported for different particle sizes (**g**) illustrates that larger particles are mostly transported toward the center of the substrate.

The PIV and FEM analysis that we have performed indicate that, in a super-hydrophobic drop, develop convective recirculating flows, which can possibly transport molecules in a suspension in different regions of the space over time (Figure 7a). Figure 7b illustrates the streamlines measured by PIV techniques in the drop, which describe how the velocity vector field varies over the drop’s domain, which is independent of time, assuming flow stationarity. The diagram in Figure 7b indicates that the fluid elements travel toward the sample substrate through straight paths in close proximity of the drop centerline, and along curved trajectories as the fluid elements approach the border. The x (perpendicular to the substrate) and y (tangent to the substrate) components of the vector field are displayed in Figure 7c and Figure 7d, respectively. For these, the maximum flow intensity is of ~360 μm/s in the direction of the substrate (x coordinate) and of ~160 μm/s in the transverse direction (y coordinate). We used these components of velocity in an algorithm that solved the Langevin equation of motion of small particulates in the drop under the influence of convection and diffusion, as described in the Methods section. We solved the equation for an equivalent time of 10 s, a sufficiently long time to let particles spread over the entire surface of the drop. In solving the equations, we noticed that the solution shows a very high sensitivity to the radius of the dislodging particles. We tested the model on two different sizes, i.e., larger particles with a diameter of 36 μm, and smaller particles with a diameter of 3 μm with a 10-fold particle diameter reduction moving from the first to the second configuration. Notably, results of the simulations indicate that larger particles are predominantly transported toward the center of the substrate, differently from smaller particles that, at the final time of the simulation, are still evenly distributed in the space, with some of the particles falling in portions of the drop distant from the substrate center (Figure 7e,f). To determine how the fluid flow characteristics affect the distribution of particles with a different size in the drop, we counted the number of particles n falling in a circle sector of radius R, central angle α=5°, and rotating by an arbitrary angle φ around the drop center. Thus, ρ=n(φ) represents the surface density of particles at the final time of the simulation as a function of the position in the drop (φ). The coordinate value φ=0 describes the center of the substrate. Values of φ larger or lower than 0 identify eccentric positions-portions of the drop increasingly distant from the center of the substrate. Figure 7g shows the density distribution ρ for (i) larger particles, (ii) smaller particles, and (iii) the superposition of the two. Diagrams in the figure indicate that, for the characteristics of flow used in the simulations, the density of particles transported toward the substrate center is larger for larger particles with a diameter of 36 μm with n~150, compared to smaller particles with a diameter of 3 μm, with n~100. Conversely, the area under the tails of the distribution is larger for smaller particles, indicating that particles with a small size are mostly transported toward the periphery of the substrate or recirculate within the drop, compared to larger particles.

## 4. Discussion

Results of the paper indicate that using a superhydrophobic surface and a temperature gradient, one can induce, within a drop, convective flows that can be tuned by changing the characteristics of the surface and the intensity of the gradient. Recent advances in nanotechnology have endowed materials scientists, engineers, and researchers with the ability to control the geometry of the surface at the micro level and nano level to obtain the wanted value of super-hydrophobicity. On the other hand, established technologies, such as plates with a tight control on the temperature or electromagnetic radiations, can generate controlled temperature patterns in a drop sitting on non-wetting surfaces. As a result, scientists have the chance to manipulate and control small amounts of liquids and solutions in un-feasible ways. The convective flow fields that emerge in the drop cause, as an effect, the locomotion of small particles dispersed therein. The characteristics of this locomotion can be deduced using the Navier–Stokes equations of viscous fluid motion and the equations of transport of substances under the influence of convection and diffusion [26,27,28,32]. Here, we have used PIV techniques to deduce the true fluid flow field developing in the drop and a numerical scheme to approximate and resolve the transport equation of particles in the system. The particle size of 10 μm that we have used was chosen to determine the field of motion within a slowly evaporating droplet. The field of motion was then used to estimate the trajectories of large (with a diameter of 36 μm) and small (with a diameter of 3 μm) particles in the drop. The comparison of theoretical predictions of the model and the equations of particle transport to the true trajectories of spherical polystyrene microbeads released in the system, determined through an imaging technique and particle tracking algorithms, shows that the results of the experiments match with the predictions of the model template with a high degree of accuracy. The numerical scheme in the formalism of Langevin equations uses successive discrete steps to represent the state of the system at a specific time and predict its evolution in the subsequent phases. Results of the simulations illustrate that the trajectories of particles in the drop show a very high sensitivity to the particles size for the same fluid-flow characteristics. Notably, larger particles (with an average diameter of 36 μm) are mostly transported toward the center of the substrate, while smaller particles (with an average diameter of 3 μm) move toward the periphery, which is far apart from the substrate center. The observation that size governs the distribution of substances in a super-hydrophobic drop suggests strategies to separate analytes in solution and resolve complex mixtures. The molecules or complexes of interest can be dispersed in a drop and progress under the effect of the convective flows described above. Then, the drop can be examined using high speed magnified imaging, fluorescence imaging, microscopy imaging, PIV, or other similar techniques. By focusing on different regions of the drop, and determining and comparing the abundance of species in those regions, researchers can estimate the fraction of low (or high) molecular weight species content of the solution. If the read-out of the experiment is sub-optimal, the researcher can potentially change parameters, such as the radius of the drop (curvature), the gradient of temperature, and the characteristics of the surface to amplify or reduce the intensity of the flow field and improve resolution. Moreover, the existence of a recirculating flow within the droplet makes the method usable for a prolonged length of time, which is quantifiable in several seconds or minutes, and only limited by the evaporation time of the drop, in which time flux differences are amplified and the performance of the device is optimized. This drop-separation scheme may achieve maximum efficiency by letting particles repeatedly circulate inside a millimeter drop. The particles are held to circular or spiral trajectories by a confining convective flux until the separation is complete. More sophisticated evolutions of this scheme that will be developed over time will integrate sensing systems, such as Raman spectroscopy or fluorescence imaging, in one chip for the complete analysis of biological solutions. The effect of size-separation resulting in the possibility of nano-chromatography has also been observed during coffee-ring formation on a wetting surface [33]. This effect is based, however, not on a recirculating flow but on mass transport to the triple contact line via Marangoni flow.

## 5. Conclusions

Superhydrophobic surfaces made of micropillars with a contact angle ≥150° induces the motion of polystyrene (PS) microparticles resembling a vertex-like motion around the center of the droplet with a maximum speed of about 0.16 mm/s observed during the first 20 min of evaporation. FEM analysis, implementing and combining heat, diffusion and Navier-Stokes equations for the considered system, shows that the motion depends on a temperature gradient along the droplet interface, which results in a shear stress $\tau_{s}$. For smaller $\tau_{s}$, a central recirculating flow turns into two symmetrical recirculating flows (Figure 6). Recirculating flows observed and numerically predicted can possibly transport molecules in a suspension in different regions of the space over time. Numerical calculations based on the Langevin equation and on the velocity flow intensity provided by FEM simulations shows a very high sensitivity to the radius of the dislodging particles. Larger particles are separately transported with respect to smaller particles. For instance, calculation on the position of the particles (during 10 s) reveals that, for the characteristics of flow used in the simulations, a bigger number of particles with a diameter of 36 μm are transported toward the substrate center. On the other hand, particles with a smaller size (3 μm) are mostly transported toward the periphery of the substrate or recirculate within the drop.

## Figures and Tables

**Table 1:** Partial differential equations, approximations, and equations after approximations.

Equation Name	Equation	Approximation	Equation after Approximation
Diffusion	$D\Delta C=\frac{\partial C}{\partial t}$	ρ>>C	ΔC=0
Navier-Stokes	$\mu \Delta \vec{v}-\nabla p-\rho \vec{v}\nabla \vec{v}-\rho \frac{\partial \vec{v}}{\partial t}=0$	Re=ρurRμ≈0	$\Delta \vec{v}=0$
Heat transfer	$\frac{\partial T}{\partial t}+\vec{v}\nabla T=k\Delta T$	$St^{-1}<<0$	ΔT=0

**Table 2:** Variables, units, and values used in numerical simulations.

Variable	Significance	Value
$\rho \;\left(g/{\mathrm{cm}}^{3}\right)$	density of liquid phase	1
μ (Pa s)	viscosity	$10^{-3}$
${\bar{v}}_{r}\left(m/s\right)$	mean velocity of the flux along the direction parallel to the substrate	$\sim 10^{-6}$
R (m)	radius of contact area of the droplet with the substrate	$\sim 10^{-3}$
Re	Reynolds number	$\sim 10^{-3}$
ϑ (°)	droplet contact angle	≥150° on SHS
$D\;\left({\mathrm{mm}}^{2}/s\right)$	diffusion coefficient in air	26.1
$C\;\left(\mathrm{mol}/m^{3}\right)$	vapor molar concentration	
Q (kJ/kg)	latent heat of water evaporation	2264
k (W/m K)	thermal conductivity	

## Data Availability

The data that support the findings of this study are available from the corresponding author upon a reasonable request.

## Supplementary Materials

The following are available online at https://www.mdpi.com/2072-666X/12/2/185/s1. See Supplementary Material for more detailed information on materials, methods, data analysis, and modeling. The video recorded with 30 frames/second shows the central circulatory flow in an evaporating droplet. Figure S1: Boundaries used for FEM simulations. (b) Schematic mesh of triangular elements for droplet and vapor phases generated by Gmsh [1]. The size of the elements is not to scale to visualize the net of connected triangles. (c) Triangular mesh covering part of the droplet with more refined elements.

## References

[1] Marinaro G. (2015). Contributions to Modeling and Applications of Superhydrophobic Surfaces for Self-Assembly of Biological Materials. Ph.D. Thesis.

[2] Marinaro G., Accardo A., Benseny-Cases N., Burghammer M., Castillo-Michel H., Cotte M., Dante S., De Angelis F., Di Cola E., Di Fabrizio E. (2014). Probing droplets with biological colloidal suspensions on smart surfaces by synchrotron radiation micro- and nano-beams. Opt. Lasers Eng..

[3] Marinaro G., La Rocca R., Toma A., Barberio M., Cancedda L., Di Fabrizio E., Decuzzi P., Gentile F. (2014). Networks of neuroblastoma cells on porous silicon substrates reveal a small world topology. Integr. Biol..

[4] Gentile F., Coluccio M.L., Coppedè N., Mecarini F., Das G., Liberale C., Tirinato L., Leoncini M., Perozziello G., Candeloro P. (2012). Superhydrophobic Surfaces as Smart Platforms for the Analysis of Diluted Biological Solutions. ACS Appl. Mater. Interfaces.

[5] Sperling M., Gradzielski M. (2017). Droplets, Evaporation and a Superhydrophobic Surface: Simple Tools for Guiding Colloidal Particles into Complex Materials. Gels.

[6] Gentile F., Coluccio M.L., Accardo A., Marinaro G., Rondanina E., Santoriello S., Marras S., Daş G., Tirinato L., Perozziello G. (2012). Tailored Ag nanoparticles/nanoporous superhydrophobic surfaces hybrid devices for the detection of single molecule. Microelectron. Eng..

[7] Marinaro G., Das G., Giugni A., Allione M., Torre B., Candeloro P., Kosel J., Di Fabrizio E. (2018). Plasmonic Nanowires for Wide Wavelength Range Molecular Sensing. Materials.

[8] Accardo A., Di Fabrizio E., Limongi T., Marinaro G., Riekel C. (2014). Probing droplets on superhydrophobic surfaces by synchrotron radiation scattering techniques. J. Synchrotron Radiat..

[9] Yang H.-Y., Moerner W.E. (2018). Resolving Mixtures in Solution by Single-Molecule Rotational Diffusivity. Nano Lett..

[10] Huang L.R., Cox E.C., Austin R.H., Sturm J.C. (2004). Continuous Particle Separation Through Deterministic Lateral Displacement. Science.

[11] Gentile F., La Rocca R., Marinaro G., Nicastri A., Toma A., Paonessa F., Cojoc G., Liberale C., Benfenati F., Di Fabrizio E. (2012). Differential Cell Adhesion on Mesoporous Silicon Substrates. ACS Appl. Mater. Interfaces.

[12] Coppedè N., Villani M., Gentile F. (2015). Diffusion Driven Selectivity in Organic Electrochemical Transistors. Sci. Rep..

[13] Gentile F., Ferrara L., Villani M., Bettelli M., Iannotta S., Zappettini A., Cesarelli M., Di Fabrizio E., Coppedé N. (2016). Geometrical Patterning of Super-Hydrophobic Biosensing Transistors Enables Space and Time Resolved Analysis of Biological Mixtures. Sci. Rep..

[14] Pradhan T.K., Panigrahi P.K. (2017). Evaporation induced natural convection inside a droplet of aqueous solution placed on a superhydrophobic surface. Colloids Surfaces A: Physicochem. Eng. Asp..

[15] Kang K.H., Lim H.C., Lee H.W., Lee S.J. (2013). Evaporation-induced saline Rayleigh convection inside a colloidal droplet. Phys. Fluids.

[16] Pan Z., Dash S., Weibel J.A., Garimella S.V. (2013). Assessment of Water Droplet Evaporation Mechanisms on Hydrophobic and Superhydrophobic Substrates. Langmuir.

[17] Adrian R.L., Adrian J., Westerweel J. (2011). Particle Image Velocimetry.

[18] Marinaro G., Accardo A., De Angelis F., Dane T., Weinhausen B., Burghammer M., Riekel C. (2014). A superhydrophobic chip based on SU-8 photoresist pillars suspended on a silicon nitride membrane. Lab Chip.

[19] Marinaro G., Burghammer M., Costa L., Dane T., De Angelis F., Di Fabrizio E., Riekel C. (2015). Directed Growth of Virus Nanofilaments on a Superhydrophobic Surface. ACS Appl. Mater. Interfaces.

[20] Thielicke W., Stamhuis E.J. (2014). PIVlab—Towards User-friendly, Affordable and Accurate Digital Particle Image Velocimetry in MATLAB. J. Open Res. Softw..

[21] Morse P.M.C., Feshbach H. (1953). Methods of Theoretical Physics.

[22] Geuzaine C., Remacle J.F. (2009). Gmsh: A Three-Dimensional Finite Element Mesh Generator with Built-In Pre- and Post-processing Facilities. Int. J. Numer. Methods Eng..

[23] Galerkin B.G. (1968). Rods and Plates: Series in Some Questions of Elastic Equilibrium of Rods and Plates.

[24] Astier Y., Datas L., Carney R., Stellacci F., Gentile F., DiFabrizio E. (2010). Artificial Surface-Modified Si3N4 Nanopores for Single Surface-Modified Gold Nanoparticle Scanning. Small.

[25] Kim M.-M. (2004). Effect of electrostatic, hydrodynamic, and Brownian forces on particle trajectories and sieving in normal flow filtration. J. Colloid Interface Sci..

[26] Gentile F., Coluccio M.L., Zaccaria R.P., Francardi M., Cojoc G., Perozziello G., Raimondo R., Candeloro P., Di Fabrizio E. (2014). Selective on site separation and detection of molecules in diluted solutions with super-hydrophobic clusters of plasmonic nanoparticles. Nanoscale.

[27] Dubin D., Diego S. (2003). Numerical and Analytical Methods for Scientists and Engineers, Using Mathematica, USA.

[28] Pearson J.E. (1993). Complex Patterns in a Simple System. Science.

[29] Hu H., Larson R.G. (2002). Evaporation of a Sessile Droplet on a Substrate. J. Phys. Chem. B.

[30] Hu H., Larson R.G. (2005). Analysis of the Microfluid Flow in an Evaporating Sessile Droplet. Langmuir.

[31] Hu H., Larson R.G. (2005). Analysis of the Effects of Marangoni Stresses on the Microflow in an Evaporating Sessile Droplet. Langmuir.

[32] Gentile F., Ferrari M., Decuzzi P. (2008). The Transport of Nanoparticles in Blood Vessels: The Effect of Vessel Permeability and Blood Rheology. Ann. Biomed. Eng..

[33] Wong T.-S., Chen T.-H., Shen X., Ho C.-M. (2011). Nanochromatography Driven by the Coffee Ring Effect. Anal. Chem..

