# Supplementary material for: Size-Exclusion Particle Separation Driven by Micro-Flows in a Quasi-Spherical Droplet: Modelling and Experimental Results
Source: Micromachines (Basel). 2021 Feb 12;12(2):185. doi: 10.3390/mi12020185 (PMC7918038; doi:10.3390/mi12020185)
Supplement: Supplementary file 1 [file micromachines-12-00185-s001.zip › Size_Exclusion_MS_SI.docx]

**Size-exclusion particle separation driven by micro-flows in a quasi-spherical droplet: modelling and experimental results**

Giovanni Marinaro ^1, 2,^ *, Christian Riekel ^3^, Francesco Gentile ^4,^ *

^1^ Institute of Process Engineering, Technische Universität Dresden, 01069 Dresden, Germany

^2^ Institute of Fluid Dynamics, Helmholtz-Zentrum Dresden-Rossendorf (HZDR), 01328 Dresden, Germany

^3^ The European Synchrotron, ESRF, CS40220, F-38043 Grenoble Cedex 9, France

^4^ Department of Experimental and Clinical Medicine, University of “Magna Graecia”, 88100 Catanzaro, Italy

* these authors share senior authorship

Authors to whom correspondence should be addressed: [francesco.gentile@unicz.it](mailto:francesco.gentile@unicz.it), [giovanni.marinaro@kaust.edu.sa](mailto:giovanni.marinaro@kaust.edu.sa)

The droplet is considered to be in an open room; however, the limit of the air domain is a spherical cap with a radius 20 times the interface radius (Ri) of the droplet (**Figure 1a,b**). We suppose that up to a distance of 20 Ri the variation of the humidity concentration as well as the temperature variation are negligible, so the humidity is kept at a value of 40% as set at the boundary of the spherical cap. Dirichlet and Neumann boundary conditions [were applied to the diffusion equation and Navier-Stokes equations](#_ENREF_5) assuming saturation vapor concentration (C_sat_) at the boundary layer and a vapor concentration of C_v_= 0.4·C_sat_ outside the boundary layer (SM Fig. 2a). Flux velocity in the droplet bulk (*v*_int_) is assumed to be due to normal and shear stresses; we also assume for the flux velocity along the substrate (*v*_sub_) the absence of fluid slip.FEM analysis was performed for a mesh of triangular elements distributed over a 5 μL droplet with Θ=155^o^ and its surrounding vapor phase (SM Fig. 2b,c). The mesh of 6152 elements across the droplet and 43854 elements for the vapor domain was created using the open source Gmsh software **(Figure 1c)**.


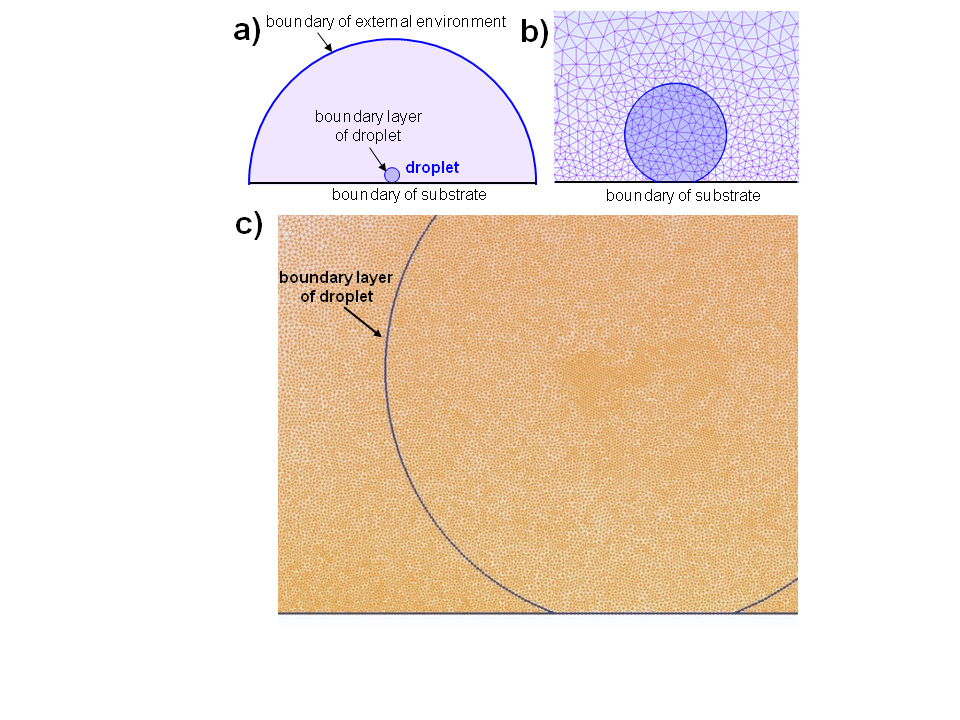


SM Figure 1. a) Boundaries used for FEM simulations. b) Schematic mesh of triangular elements for droplet and vapor phases generated by Gmsh ^1^. The size of the elements is not to scale to visualize the net of connected triangles. c) Triangular mesh covering part of the droplet with more refined elements.


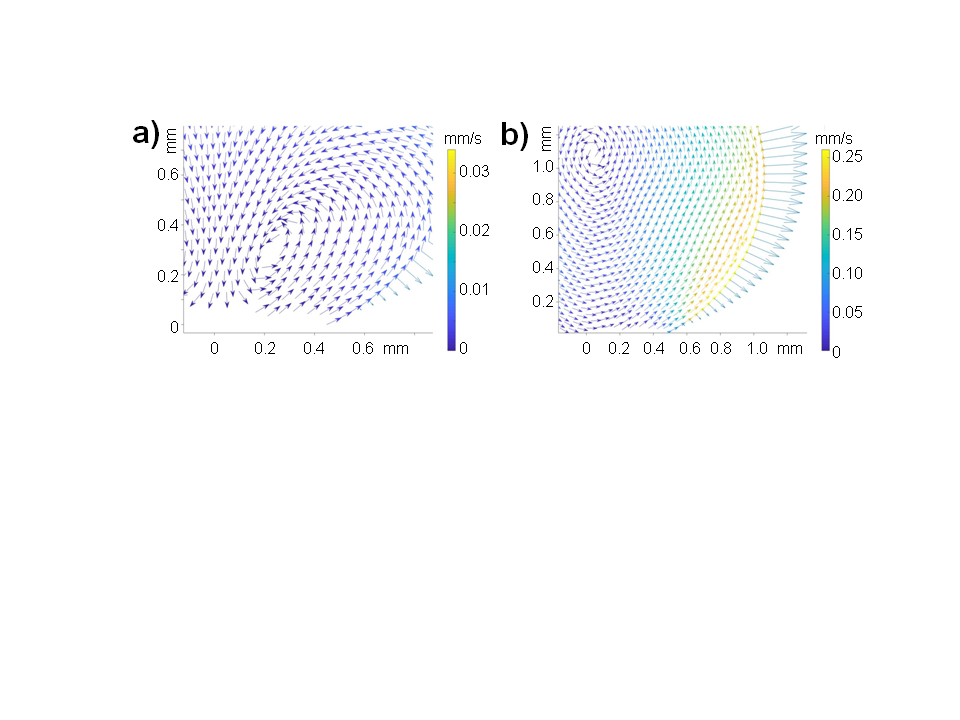


SM Figure 2. a) Zoom of vector map in Fig. 3e for recirculating flows in the two hemispheres of a droplet. b) Zoom of vector map for central recirculating flow. Simulations for 21^o^C and 40 % humidity.

1. Geuzaine, C. & Remacle, J. F. Gmsh: a Three-Dimensional Finite Element Mesh Generator with Built-In Pre- and Postprocessing Facilities. *International Journal for Numerical Methods in Engineering* **79**, 1309–1331 (2009).
